# Measurement of complex optical susceptibility for individual carbon nanotubes by elliptically polarized light excitation

**DOI:** 10.1038/s41467-018-05932-9

**Published:** 2018-08-23

**Authors:** Fengrui Yao, Can Liu, Cheng Chen, Shuchen Zhang, Qiuchen Zhao, Fajun Xiao, Muhong Wu, Jiaming Li, Peng Gao, Jianlin Zhao, Xuedong Bai, Shigeo Maruyama, Dapeng Yu, Enge Wang, Zhipei Sun, Jin Zhang, Feng Wang, Kaihui Liu

**Affiliations:** 1 State Key Laboratory for Mesoscopic Physics, Collaborative Innovation Centre of Quantum Matter, School of Physics, Peking University, Beijing, 100871 China; 2 Center for Nanochemistry, College of Chemistry and Molecular Engineering, Peking University, Beijing, 100871 China; 3 School of Science, Northwestern Polytechnical University, Xi’an, 710129 China; 4 International Center for Quantum Materials and Electron Microscopy Laboratory, Peking University, Beijing, 100871 China; 5 Institute of Physics, Chinese Academy of Sciences, Beijing, 100875 China; 6 Department of Mechanical Engineering, The University of Tokyo, Tokyo, 113-8656 Japan; 7 Energy NanoEngineering Lab, National Institute of Advanced Industrial Science and Technology, Tsukuba, 305-8564 Japan; 8 Department of Physics, Southern University of Science and Technology, Shenzhen, 518055 China; 9 Department of Electronics and Nanoengineering, Aalto University, Espoo, 02150 Finland; 10 QTF Centre of Excellence, Department of Applied Physics, Aalto University, Espoo, 02150 Finland; 11 Department of Physics, University of California at Berkeley, Berkeley, CA 94720 USA

## Abstract

The complex optical susceptibility is the most fundamental parameter characterizing light-matter interactions and determining optical applications in any material. In one-dimensional (1D) materials, all conventional techniques to measure the complex susceptibility become invalid. Here we report a methodology to measure the complex optical susceptibility of individual 1D materials by an elliptical-polarization-based optical homodyne detection. This method is based on the accurate manipulation of interference between incident left- (right-) handed elliptically polarized light and the scattering light, which results in the opposite (same) contribution of the real and imaginary susceptibility in two sets of spectra. We successfully demonstrate its application in determining complex susceptibility of individual chirality-defined carbon nanotubes in a broad optical spectral range (1.6–2.7 eV) and under different environments (suspended and in device). This full characterization of the complex optical responses should accelerate applications of various 1D nanomaterials in future photonic, optoelectronic, photovoltaic, and bio-imaging devices.

## Abstract (web summary)

One-dimensional materials such as carbon nanotubes have many applications, but not all of their properties can be described in the same way as for conventional media. Here, the authors devise a method to measure the complex optical susceptibility in a 1D nanomaterial and demonstrate it for carbon nanotubes.

## Introduction

One-dimensional (1D) materials are at the center of significant research effort of nano science and technology. For example, carbon nanotubes, a 1D material from rolled-up graphene, have shown fascinating optical properties, e.g., quantized optical transitions^1–3^, strong many-body interactions^4–9^ and efficient photon-electron generation^10^, thus enabling diverse applications ranging from photonics^11–14^, optoelectronics^15–17^, and photovoltaics^18^ to bio-imaging^19^. To fully utilize 1D materials for various application, we need to know their optical response parameters that quantitatively describe light-matter interactions, among which the complex optical susceptibility ($\tilde{\chi}$) is the most fundamental one^20,21^ (describing the response of the dipole moment **P** of crystalline materials to external optical field **E**, **P** = $\tilde{\chi}$ *ε*_0_**E**, $\tilde{\chi}$ =$\tilde{\epsilon}$–1). Unlike mature technology for measuring the complex optical susceptibility by solving Fresnel equations in two-dimensional (2D) and three-dimensional (3D) materials^22^, there is no available technique to effectively measure both the real and imaginary parts of $\tilde{\chi}$ in individual 1D materials. The main difficulty lies in the vanishing of concept for coherent refraction or reflection and thus conventional methodologies become invalid^23^. One way to circumvent this difficulty is to measure the absorption of 1D materials (e.g., carbon nanotubes^24–26^), which is proportional to the imaginary part susceptibility (*χ*_2_). By employing the Kramers–Kronig (K-K) relation, one can in principle calculate the real part susceptibility *χ*_1_. However, to get the accurate value, the application of K-K relation requires experimental data of *χ*_2_ over an extremely broad energy region from 0 eV to infinity, which is never available. Until now, there is no method to directly measure the complex optical susceptibility of individual 1D materials.

Here we develop a methodology to measure the complex optical susceptibility for individual carbon nanotubes by an elliptical-polarization-based optical homodyne detection. By accurately controlling the interference between incident left-handed and right-handed elliptical polarization beam and nanotube-scattering light, we obtain two sets of optical spectra containing both *χ*_1_ and *χ*_2_ information with different pre-coefficients, which allow us to determine the quantitative value of $\tilde{\chi}$. In addition, our technique also enhances the optical signal level by about two orders of magnitude, making the extremely weak individual nanotube signal readily detectable in a broad optical spectral range (1.6–2.7 eV) and in different environments (suspended and in device). Our results can open up exciting opportunities in characterizing a variety of 1D nanomaterials, including graphene nanoribbon, nanowires, and other nano-biomaterials, thus facilitating their accurate material design and applications in future photonic, optoelectronic, photovoltaic, and bio-imaging devices.

## Results

### Scheme of complex optical susceptibility measurement in a transmission geometry

For individual nanotubes with diameter (~1 nm) much less than light wavelength (~ 500 nm), their optical signal in reflection/transmission geometry can be viewed as the interference between optical reference and nanotube-scattering fields^27,28^. To obtain both *χ*_1 _and *χ*_2 _of a nanotube, in principle we need two optical reference components with a π/2 phase difference. Actually, circularly/elliptically polarized light naturally provides such a π/2 phase difference, which has been proved to be an effective excitation for optical tomography^29^, spin/quantum computing and information^30^, valleytronics^31^, and high-harmonic generation^32^. In our work, we choose elliptical polarization light with a relatively large ellipticity as excitation. Its advantage over circularly polarized light lies in that the reference beam can be greatly reduced by two vertically placed polarizers, which will greatly enhance the final optical contrast as demonstrated in the previous single-tube Rayleigh scattering, absorption and reflection measurement using linear polarization excitation light^26,33,34^.

**Fig. 1:**
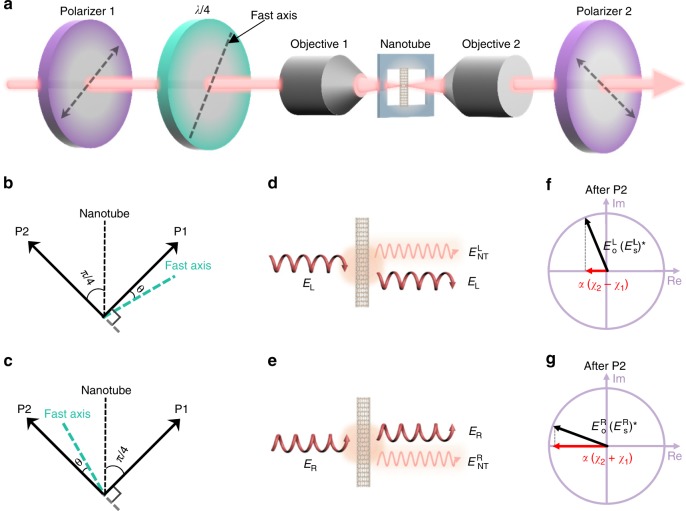
Scheme of complex optical susceptibility measurement in a transmission geometry. **a** Scheme of experiment setup. Two polarizers were strictly perpendicular to each other. A quarter-wave plate was used to generate the elliptical chirality (left- or right-handed). A vertically placed carbon nanotube was put at the focus of the two confocal objectives. **b**, **c** Layouts of the polarization control and the nanotube. The nanotube was laid at the bisector of the two polarizers’ axis. The fast axis of the wave plate was kept at a small angle (*θ*) to the polarizer axis. Polarizer 2 and polarizer 1 were abbreviated to P2 and P1. **d**, **e** Interference scheme of input left- (*E*_L_)and right-handed (*E*_R_)elliptically polarized light and nanotube forward-scattering field ($E_{{\mathrm{NT}}}^{\mathrm{L}}$ or $E_{{\mathrm{NT}}}^{\mathrm{R}}$). **f**, **g** Illustrations of complex phase diagrams of the detected optical contrast signal with left/right elliptically polarized light excitation after polarizer 2, in which *χ*_1_ contributes oppositely under two layouts

We first apply this technique to individual suspended single-walled carbon nanotubes in a transmission geometry. Experimental scheme of our elliptical-polarization-based optical homodyne detection method is shown in Fig. 1a. A supercontinuum laser was used as the light source to provide a broadband excitation (450–900 nm), a pair of confocal polarization-maintaining objectives served to focus the supercontinuum light on individual nanotubes and collected the transmitting and nanotube-scattering lights, two polarizers and a quarter-wave plate were used to generate elliptically polarized light and control the polarization of transmitted light. Here we should note that the very careful selection of objectives and wave plate to maintain the polarization purity is crucial to realize the final signal detection (See optical component details in Methods). Layouts of the polarization control are shown in Fig. 1b, c. The two polarizers were set strictly perpendicular to each other, and the suspended nanotube was positioned at an angle of π/4 with respect to two polarizers. In the two different configurations, fast axis of the quarter-wave plate was kept at a small angle (*θ*) with polarizer 1 (Fig. 1b) or polarizer 2 (Fig. 1c) to generate left-handed (***E***_**L**_) or right-handed (***E***_**R**_) elliptical-polarization light as the excitation for the nanotube. Due to the very strong 1D depolarization effect, the nanotube-scattering field is mainly polarized along the nanotube axial direction^35,36^. Therefore, the nanotube forward-scattering field can be written as $E_{\mathrm{NT}}^{\mathrm{L}}=\beta \tilde\chi E_{\mathrm{L}}$ and $E_{\mathrm{NT}}^{\mathrm{R}}=\beta \tilde\chi E_{\mathrm{R}}$, respectively, where *β* represents a scattering coefficient. Before polarizer 2, the forward-scattering light of the nanotube interferes with the transmitted optical reference beam (Fig. 1d, e), which yields the optical contrast signal as

1 $$\frac{{\Delta T_{\mathrm{i}}}}{T} = \frac{{\left| {E_{\mathrm{i}} + E_{{\mathrm{NT}}}^{\mathrm{i}}} \right|^2 - \left| {E_{\mathrm{i}}} \right|^2}}{{\left| {E_{\mathrm{i}}} \right|^2}} \approx \frac{{2{\mathrm{Re}}\left( {E_{\mathrm{i}}E_{{\mathrm{NT}}}^{{\mathrm{i}} \ast }} \right)}}{{\left| {E_{\mathrm{i}}} \right|^2}},$$

where *T*_i_ is the transmission signal intensity, Δ*T*_i_ is the change of transmission signal intensity resulted from the presence of a nanotube, and i stands for L or R (The $|E_{{\mathrm{NT}}}^{\mathrm{i}}|$^2^ term has been neglected because it is orders of magnitude smaller than the cross term). After polarizer 2, the reference light field is decreased dramatically due to small eccentricity of elliptically polarized light $\left( {E_{\mathrm{o}}^{\mathrm{i}} = \left( {E_{\mathrm{i}}\sin 2\theta } \right)/\sqrt 2 } \right)$, while the nanotube’s scattering field is only decreased by a small proportion $(E_{\mathrm{s}}^{\mathrm{i}} = E_{{\mathrm{NT}}}^{\mathrm{i}}/\sqrt 2 )$. Therefore, the optical contrast is greatly enhanced, typically by 20 times in our transmission geometry. More importantly, the variation of the modulated signals depends on the real part of the cross term $E_{\mathrm{o}}^{\mathrm{i}}(E_{\mathrm{s}}^{\mathrm{i}})^ \ast$, and this term varies with the elliptical chirality of incident light (Fig 1f, g). In detail, detected optical contrast signals caused by left- or right-handed incident light can be written as:

2 $$\frac{{\Delta T_{\mathrm{L}}}}{T} = \alpha \left( {\chi _2 - \chi _1} \right),\frac{{\Delta T_{\mathrm{R}}}}{T} = \alpha \left( {\chi _2 + \chi _1} \right),\alpha = \frac{\beta }{{{\mathrm{sin}}2\theta }}.$$

Since *χ*_1_ and *χ*_2_, respectively, have the opposite and same contribution in the optical signal with left- and right-handed elliptically polarized excitation, the solution of both *χ*_1_ and *χ*_2_ can be obtained unambiguously (See more details in Supplementary Note 1).

### Complex optical susceptibility measurement of individual suspended nanotube

**Fig. 2:**
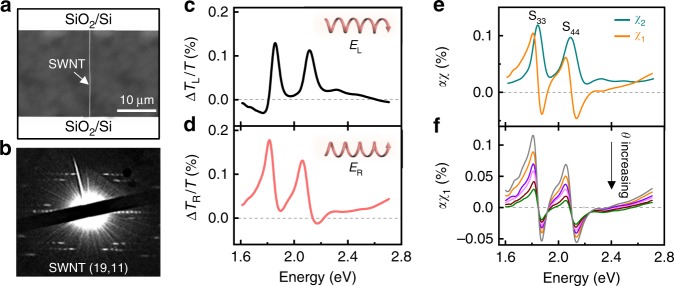
Complex optical susceptibility measurement of individual suspended nanotube. **a** Scanning electron micrograph (SEM) image of a suspended carbon nanotube across an open slit etched on SiO_2_/Si substrate. **b** The electron diffraction pattern reveals the chiral index of nanotube as (19, 11), a semiconducting tube with a diameter of 2.06 nm. **c**, **d** Optical contrast spectra with left- (*E*_L_) (**c**) and right-handed (*E*_R_) (**d**) elliptically polarized excitation. The angle *θ* between the wave plate and polarizer 1 or polarizer 2 is set as 2°. **e** Real (*αχ*_1_, orange line) and imaginary (*αχ*_2_, green line) susceptibility of the nanotube under *θ* = 2°. The two peaks are corresponded to S_33_ and S_44_ optical transitions, respectively. **f** Dependence of detected real susceptibility value (*αχ*_1_) on *θ*. With *θ* increasing from 1.4° to 4°, the signal decreases linearly with 1/sin2*θ* (Supplementary Fig. 1). *α* is a detection coefficient

Scanning electron microscopic image of a nanotube suspended across an open slit in SiO_2_/Si substrate is shown in Fig. 2a. The chiral index of this nanotube was identified as (19, 11) from its electron diffraction pattern (Fig. 2b) and its Rayleigh scattering or absorption spectrum^37^. Figure 2c, d shows optical contrast signals in photon energy range of 1.60–2.70 eV under left- (Fig. 2c) and right-handed (Fig. 2d) elliptically polarized light excitation (*θ* = 2°). This set of spectra show two characteristic features, which are significantly different from absorption spectra^26^: (1) the peaks are not Lorentzian like, with slow bumps and pits; (2) optical contrast signal goes below zero at some energies. These features are originated from the opposite (same) contribution of *χ*_1_(*χ*_2_) to the optical signal. According to Eq. 2, we extract the spectra of *χ*_1_ (orange line) and *χ*_2_ (green line) separately (Fig. 2e). The two optical resonances sit at 1.83 and 2.09 eV, corresponding to S_33_ and S_44_ optical transitions of the nanotube. The main peaks of *χ*_2_ can be decomposed into the dominant exciton contribution and a continuum contribution (band-to-band transitions)^26^. The weak peak located at 2.3 eV above the main resonance is attributed to a phonon side band^38–41^. In addition to solving *χ*_1 _and *χ*_2_, the elliptical polarization excitation also enables the enhancement of the detected contrast signal as we accurately control the fast axis angle *θ*. When *θ* increases from 1.4° to 4°, the detected signal (taking *αχ*_1_ as an example) decreases linearly with 1/sin2*θ* (Fig. 2f and Supplementary Fig. 1), which is in accordance with the quantitative analysis of Eq. 2.

### Systematic complex optical susceptibility measurement of individual nanotubes

**Fig. 3:**
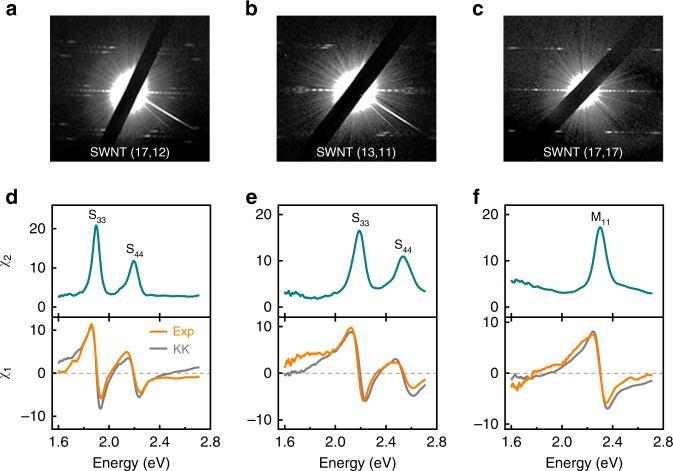
Systematical complex optical susceptibility measurement of individual nanotubes. **a**–**c** Electron diffraction patterns of three single-walled carbon nanotubes with different chiral indices. The (17, 12) semiconducting nanotube (**a**) has a diameter of 1.98 nm; the (13,11) semiconducting nanotube (**b**) has a diameter of 1.63 nm; the (17,17) metallic nanotube (**c**) has a diameter of 2.31 nm. **d**–**f**, Measured imaginary (*χ*_2_, green) and real (*χ*_1_, orange) susceptibility of nanotubes in **a**–**c** under *θ* = 2°. Optical transitions types are marked above each peak. The calculated real susceptibility ($\chi _1^{{\mathrm{KK}}}$, gray) through Kramers–Kronig transformation of *χ*_2_ in a finite photon energy range (1.6–2.7 eV) were also shown. A good agreement between *χ*_1_ and $\chi _1^{{\mathrm{KK}}}$ is achieved around the resonance peak region, while obvious deviation can happen in non-resonant region. Optical transitions are marked above each peak

By estimating the scattering coefficient *β* in our experiment (See Supplementary Note 2), we can further give out the absolute value of $\tilde{\chi}$ for a nanotube. In total, 20 single-walled carbon nanotubes are measured (Fig. 3 and Supplementary Fig. 2 and Fig. 3) and three representative complex susceptibilities of them are shown in Fig. 3. From the electron diffraction patterns and optical transitions (Fig. 3a–c), these three nanotubes are identified as semiconducting (17, 12) and (13, 11) and metallic (17, 17), respectively. Obviously, both *χ*_2_ and *χ*_1_ spectra (Fig. 3d–f) are intrinsic characteristics of a nanotube. As the ultralong nanotubes on open slits are typically with diameters of 1.5–3.0 nm, we can only access optical transitions higher than S_22_ in our laser spectral range of 1.6–2.7 eV. In principle, we can also measure E_11_ transition as long as one can grow the ultralong nanotubes with diameter less than 0.8 nm on the wide slit in the future.

According to Kramers–Kronig relation, the *χ*_1_ can be obtained from *χ*_2_ by

3 $$\chi _1\left( E \right) = \frac{2}{{\mathrm{\pi }}}{\int}_0^\infty {\frac{{E^\prime \chi _2\left( {E^\prime } \right)}}{{(E^\prime )^2 - E^2}}} dE^\prime .$$

This integration requires the full energy data ranging from 0 eV to infinity of *χ*_2_. Nevertheless, by utilizing the measured *χ*_2_ data in energy range of 1.6–2.7 eV to perform K-K transformation, we can deduce a predicted $\chi _1^{{\mathrm{KK}}}$ (Fig. 3d–f, gray lines). We found a relative good agreement between *χ*_1_ and $\chi _1^{{\mathrm{KK}}}$ around the resonance peak region, while obvious deviations in non-resonant region were observed. The agreement in the resonant region actually proves the accuracy of our measurement of *χ*_1_, because the denominator weight factor around resonance region in Eq. 3 (*E*^'^ is close to *E*) is very large and the ignorance of other non-resonant regions should still yield pretty good prediction. The agreement also implies the conventional susceptibility model describing optical responses still works even for such 1D nanostructures with many molecule-like optical properties. While the deviation in the non-resonant region highlights the necessity of independent *χ*_1_ measurement other than predicted $\chi _1^{{\mathrm{KK}}}$ since one could never obtain *χ*_2_ in full energy region. The discrepancy can be understood from Eq. 3. When conducting K-K transformation in a limited range of 1.6–2.7 eV, the influence of *χ*_2_ spectra outside the range is not considered, resulting in an inaccurate calculated value of $\chi _1^{{\mathrm{KK}}}$. *χ*_2_ below 1.6 eV gives a negative contribution to calculated $\chi _1^{{\mathrm{KK}}}$, while *χ*_2_ above 2.7 eV gives a positive contribution. Therefore, the accurate distribution of those unconsidered optical transitions mainly determines the deviation between *χ*_1_ and $\chi _1^{{\mathrm{KK}}}$. In particular, the optical transitions are fingerprints of each nanotube with different chirality, therefore a simple K-K calculation from *χ*_2_ of limited energy range in principle cannot produce accurate $\chi _1^{{\mathrm{KK}}}$ constantly.

### On-chip complex optical susceptibility detection of individual nanotubes

**Fig. 4:**
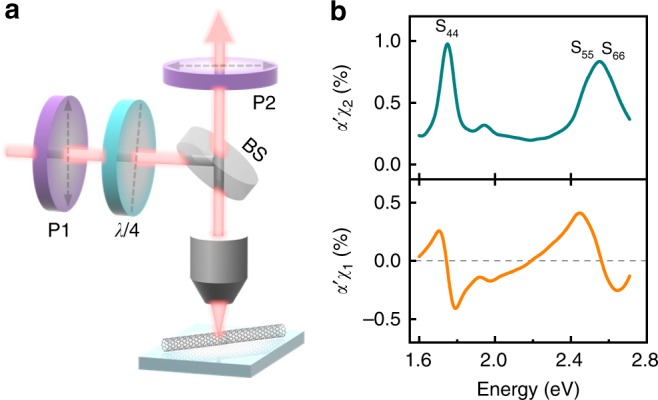
On-chip complex optical susceptibility detection of individual nanotubes. **a** Scheme of the experiment setup in the reflection configuration. Beam splitter was abbreviated to BS. **b** Imaginary (*χ*_2_, green) and real (*χ*_1_, orange) susceptibilities of nanotube (25,11) on fused quartz substrate. *α*' is a detection coefficient. Optical transitions are marked above each peak. S_55_ and S_66_ transitions are very close with each other

As for most optical applications in devices, nanotubes will be on substrates. Furtherly, we developed our technique for nanotubes on substrates in a reflection geometry (Fig. 4a). Compared with the transmission geometry, a reflection pre-factor (1 + *r*)^2^/*r* is added to final contrast signal (*α*′ = *α*(1 + *r*)^2^/*r*), where *r* is the reflection coefficient calculated from Fresnel equations and 1 + *r* is the local field experienced by the nanotube (See more details in Supplementary Note 3). This will lead to even higher contrast signal enhancement to ~100 times, in contrast to the ~20 times enhancement for suspended nanotubes in the transmission geometry. In Fig. 4b, we show a representative measurement on semiconducting (25, 11) nanotubes with nice *χ*_1_ and *χ*_2_ spectra. Optical transition peaks identified from the extracted imaginary data were used to determine the nanotube chirality based on the atlas developed before^37^. Same as transmission spectrum, deduced $\chi _1^{{\mathrm{KK}}}$ agrees with the experimental value around the resonance peak region (See Supplementary Fig. 4).

## Discussion

An advantage of carbon nanotubes for optical applications is the well-defined atomic structure and associated optical transitions that can be accurately described by both theory and experimental data bases^37^. From our $\tilde{\chi}$ measurement, we can clearly see that every nanotube has different *χ*_1_ and *χ*_2_ value at different energy regions, which is a great advantage in the nanophononics, including but not limited to metasurfaces. With the spatial control of the $\tilde{\chi}$-determined nanotubes, one can readily tailor the wave fronts of light beams with arbitrary phase, polarization and amplitude distributions, which holds a great promise to implement the subwavelength optical components^42^, optical computation^43^, and information processing^44^, while enjoying the merits of the low loss and reduced dimension because the 1D nature of nanotubes. Further, nanotubes can have different walls, such as single wall and double walls with more optical transitions (See Supplementary Fig. 5), and the richness and flexibility in the optical engineering for meta-materials applications are therefore very promising^45^.

In summary, the direct measurement of the basic complex optical susceptibility of 1D materials is obviously fundamental for their accurate design and applications in the future photonic, optoelectronic, photovoltaic and bio-imaging devices, provides a new detectable parameter to monitor the external regulations such as charge doping, strain, molecular adsorption, and dielectric environment, and will further evoke theoretical understanding of 1D materials by complex susceptibility. The signal detection limit of our technique is estimated to be ~10^−6^ (See Supplementary Note 4). This high sensitivity can ensure the detection of nanotubes with the smallest diameter down to 0.3 nm. Thus, for general 1D materials with defined structure, such as long graphene nanoribbons and semiconductor nanowires, our technique is expected to work as well.

## Methods

### Nanotube preparation

In this study, nanotubes were grown by chemical vapor deposition method. We used ethanol through argon bubble as carbon precursor and a thin ion film (0.2 nm) as catalyst for the synthesis at 950 °C for 30 min. Suspended SWNTs were grown across open slit structures on SiO_2_/Si substrate.

### TEM (Transmission Electron Microscope) characterization

Electron diffraction patterns were obtained in TEMs (JEOL 2010F and JEM ARM 300CF) operated at 80 kV. Both electron beam and laser beam can go through the open slit with individual carbon nanotubes, which enables directly investigation of the chiral indices and optical spectrum of the same nanotube.

### Optical setup

For transmission configuration (Fig. 1a), optical signal was collected by a home-built confocal microscope system, where a supercontinuum laser (Fianium SC-400-4) is used as the light source, shooting light through polarizer 1 (Thorlabs, GTH10M) and a quarter-wave plate (Thorlabs, AQWP05M-600A). Then an objective (Mitutoyo M Plan 50 X, NA = 0.42) serves to focus the light to the sample and another objective (Mitutoyo M Plan 50 X, NA = 0.42) collects the transmitted light. An oblique objective (Mitutoyo M Plan 50 X, NA = 0.42) was used to collect nanotube’s scattering signal to a CCD camera (PULNIX TM-7CN) for Rayleigh imaging. The transmission signal was modulated by polarizer 2 (Thorlabs, GTH10M). Two sets of spectra with the nanotube in and out of the beam focus were obtained to generate the contrast spectra by a spectrometer containing a grating (Thorlabs, GT50-03) and a linear CCD (Imaging Solution Group, LW ELIS-1024a-1394). The Gaussian spatial profile of the light source was experimentally mapped by gradually changing the distance between the carbon nanotube and the beam focus (Supplementary Fig. 6). In the reflection geometry (Fig. 4a), the main difference is the use of a beam splitter (customer-polished quartz glass) and only one objective (Nikon S Plan Fluor 40 X, NA = 0.65).

### Data availability

The authors declare that all relevant data are included in the paper and its Supplementary Information files. Additional data are available from the corresponding author upon reasonable request.

## Electronic supplementary material

Supplementary Information
