# Supplementary material for: Measurement of complex optical susceptibility for individual carbon nanotubes by elliptically polarized light excitation
Source: Nat Commun. 2018 Aug 23;9:3387. doi: 10.1038/s41467-018-05932-9 (PMC6107641; doi:10.1038/s41467-018-05932-9)
Supplement: Supplementary file 1 — Supplementary Information [file 41467_2018_5932_MOESM1_ESM.pdf]

Supplementary Information

Measurement of Complex Optical Susceptibility for Individual Carbon Nanotubes by Elliptically Polarized Light Excitation

Fengrui Yao, Can Liu, Cheng Chen, Shuchen Zhang, Qiuchen Zhao, Fajun Xiao, Muhong Wu, Jiaming Li, Peng Gao, Jianlin Zhao, Xuedong Bai, Shigeo Maruyama, Dapeng Yu, Enge Wang, Zhipei Sun, Jin Zhang, Feng Wang and Kaihui Liu*

The supplementary information includes:

Supplementary Figure 1-6

Supplementary Note 1-4

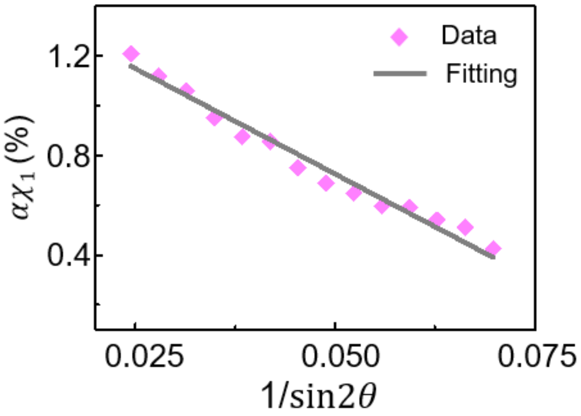

Supplementary Figure 1. Relationship between the detected contrast signal and angle $\boldsymbol{\theta}$.

The detected signal ($\alpha\chi_1$) is linear dependent on $1/\sin 2\theta$ at photon energy of 1.81 eV.

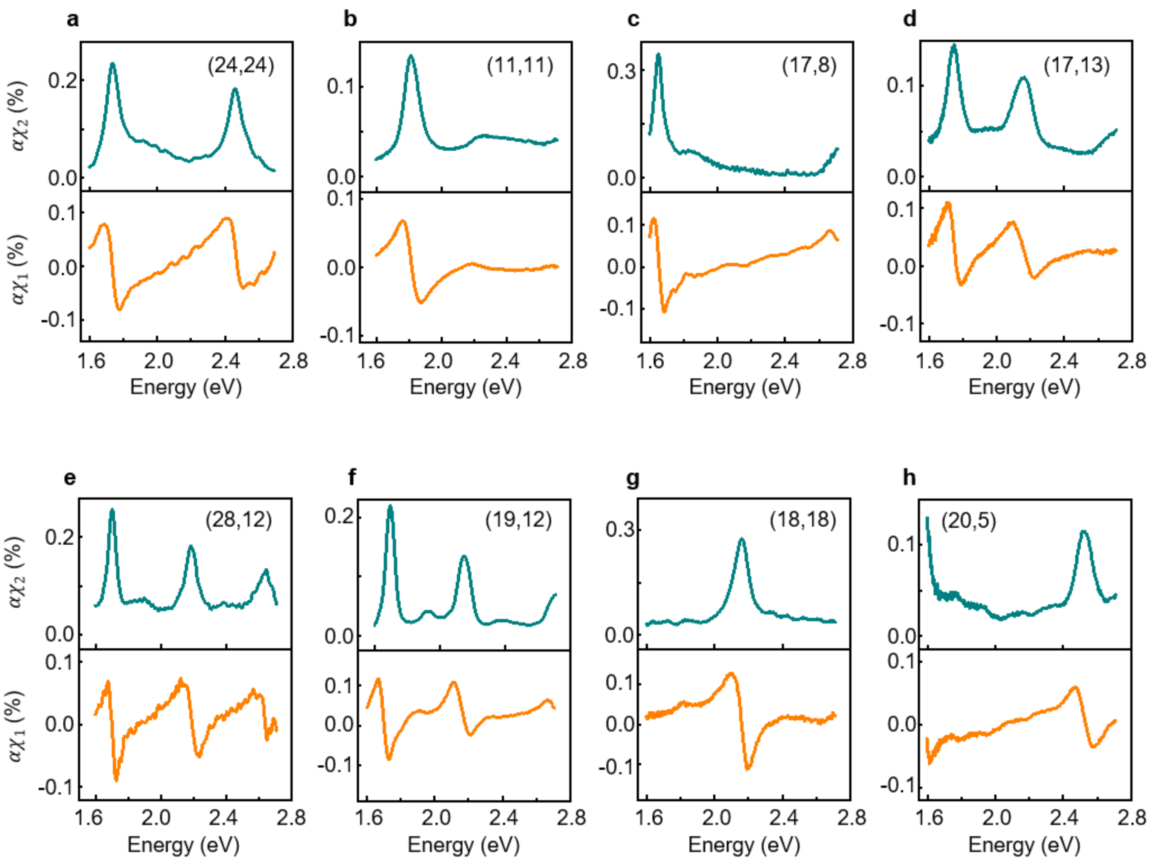

Supplementary Figure 2. Measured complex susceptibility of 8 SWNTs with different chiral indices. Imaginary (χ_2, green) and real (χ_1, orange) were show seperately. Determination of chirality was based on the electron diffraction pattern or the atlas of nanotube optical transitions[1]. The accuracy of the mesurement was further confirmed by converting the measured imaginary susceptiblity data to absolute absorption cross-sections, the average value of which converges on the graphene value[2].

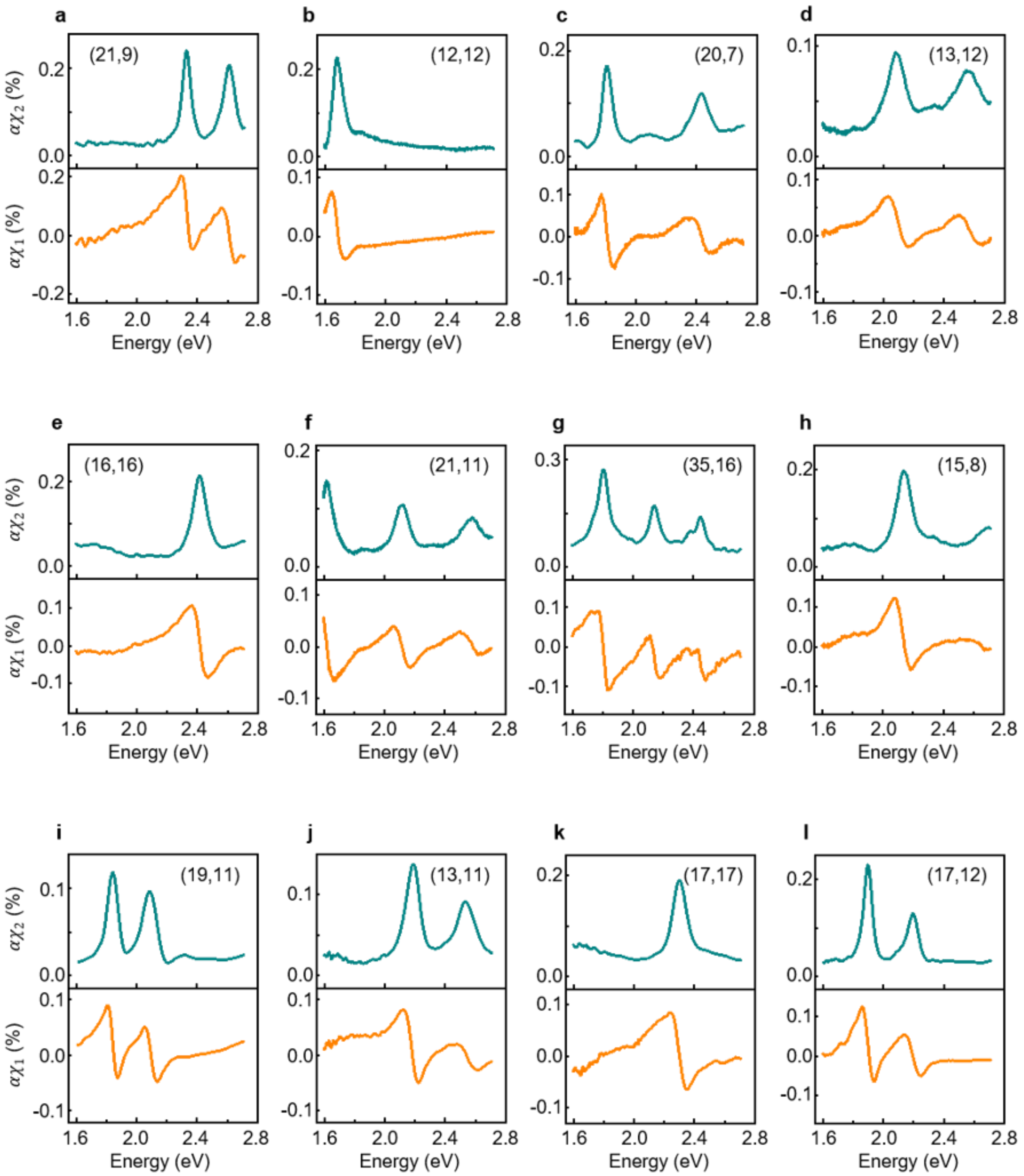

Supplementary Figure 3. Measured complex susceptibility of 12 SWNTs with different chiral. Imaginary (χ_2, green) and real (χ_1, orange) were show seperately. Determination of chirality is the same as data in Supplementary Fig. 2.

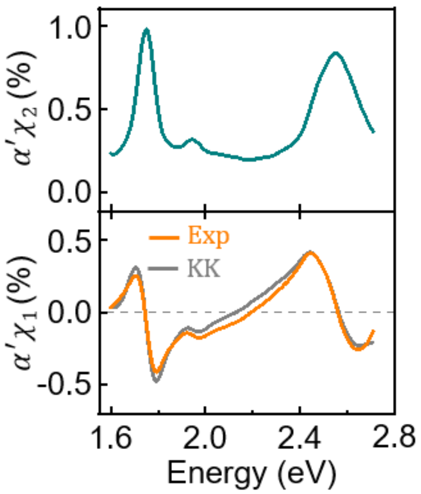

Supplementary Figure 4. Contrast of experimental real susceptibilities and simulated data. Imaginary (χ_2, green) and real (χ_1, orange) susceptibilities of nanotube (25,11) on fused quartz substrate. α' is a detection coefficient. The calculated real susceptibility (χ_1^{KK}, gray) through Kramers-Kronig transformation of χ_2 in a finite photon energy range (1.6-2.7 eV) was also shown.

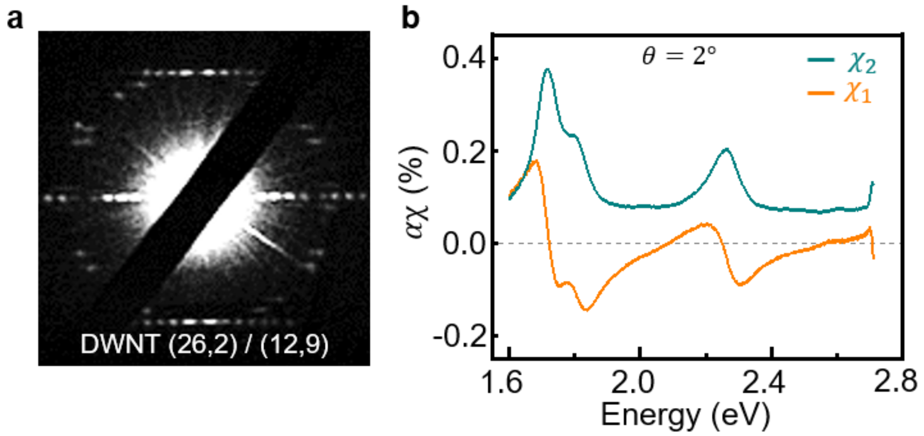

Supplementary Figure 5. Complex optical susceptibility measurement of individual double-walled carbon nanotube (DWNT). a, Electron diffraction pattern of DWNT (26,2)/(12,9). The outer (inner) tube is metallic with a diameter of 2.12 (1.43) nm. **b**, Complex susceptibility of the same DWNT. The two resonances at 1.68 and 1.72 eV of χ_2 (green line) correspond to the M_{11}^{-} and M_{11}^{+} electronic transitions of outer tube (26, 2), and the resonances at 2.20 eV correspond to the M_{22}^{-} electronic transitions of inter tube (12, 9)[1]. The angle θ between the waveplate and polarizer 1 or polarizer 2 is kept at 2° for this measurement.

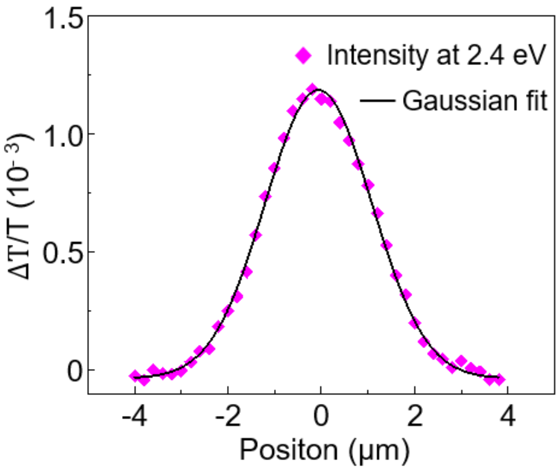

Supplementary Figure 6. Gaussian beam shape. Dependence of contrast signal at 2.4 eV on nanotube's position in the beam focus and its fitting to Gaussian function of Supplementary Equation 8.

Supplementary Note 1. Extract real and imaginary part of complex optical susceptibility from the transmission homodyne modulation signal.

In the first case, to generate a left-handed elliptically polarized light, we kept the angle between the quarter-wave plate and the first polarizer (P1) at θ. Let E_{in} denote the electric field of the incident light after P1. Modulated by a quarter-wave plate, the local field experienced by the nanotube in this case would be left-handed, with the expression: $\mathbf{E}_{\mathrm{L}} = E_{\mathrm{in}}\mathrm{e}^{-\mathrm{i}\frac{\pi}{2}}\cos\theta\mathbf{i} + E_{\mathrm{in}}\sin\theta\mathbf{j}$, where $\mathbf{i}$ and $\mathbf{j}$ are unit vectors along the fast and slow axis of the quarter-wave plate, respectively, and $\mathrm{e}^{-\mathrm{i}\frac{\pi}{2}}$ denotes the phase difference between the fast and slow axis of the quarter-wave plate. The first term is the electric field along the fast axis, while the second term is that along the slow axis. Then the transmitting light after the second polarizer (P2) ($E_{\mathrm{o}}^{\mathrm{L}}$) is

$$E_{\mathrm{o}}^{\mathrm{L}} = \mathrm{e}^{-\mathrm{i}\frac{\pi}{2}}E_{\mathrm{in}}(\mathrm{e}^{-\mathrm{i}\frac{\pi}{2}}\cos\theta\sin\theta - \sin\theta\cos\theta) = (\mathrm{i}-1)E_{\mathrm{in}}\sin\theta\cos\theta. \quad (1)$$

Where the first $e^{-\mathrm{i}\frac{\pi}{2}}$ term is caused by Gouy's phase shift, and the second $e^{-\mathrm{i}\frac{\pi}{2}}$ term is from the phase difference between the fast and slow axis of a quarter-wave plate. The nanotube scattering electric field is polarized along the nanotube direction due to its strong depolarization effect, the scattering field (E_{NT}) amplitude would be

$$E_{\mathrm{NT}}^{\mathrm{L}} = \beta\tilde{\chi}E_{\mathrm{L}} = \beta\tilde{\chi}E_{\mathrm{in}}\left[\mathrm{e}^{-\mathrm{i}\frac{\pi}{2}}\cos\theta\cos\left(\frac{\pi}{4}+\theta\right) + \sin\theta\cos\left(\frac{\pi}{4}-\theta\right)\right]. \quad (2)$$

Here β represents an efficiency coefficient (details in Supplementary Note 2). Then, we could derive the nanotube scattering field after P2 ($E_{\mathrm{s}}^{\mathrm{L}}$) as

$$E_{\mathrm{s}}^{\mathrm{L}} = \beta\tilde{\chi}E_{\mathrm{in}}\left[\mathrm{e}^{-\mathrm{i}\frac{\pi}{2}}\cos\theta\cos\left(\frac{\pi}{4}+\theta\right)\cos\frac{\pi}{4} + \sin\theta\cos\left(\frac{\pi}{4}-\theta\right)\cos\frac{\pi}{4}\right]. \quad (3)$$

Based on supplementary equations (1) and (3), we could derive that:

$$\frac{\Delta T_{\mathrm{L}}}{T}=\frac{2\mathrm{Re}\left(E_{\mathrm{o}}^{\mathrm{L}}(E_{\mathbf{s}}^{\mathrm{L}})^{*}\right)}{\left|E_{\mathrm{o}}^{\mathrm{L}}\right|^{2}}=\frac{\beta\chi_{2}(\cos2\theta-\sin2\theta)}{\sin2\theta}-\frac{\beta\chi_{1}}{\sin2\theta}\approx\frac{\beta(\chi_{2}-\chi_{1})}{sin2\theta}. \quad (4)$$

The approximation at last is reasonable because θ is set as a small angle ($\theta \leq 4°$).

In the second case, to generate a right-handed elliptically polarized light, we keep the angle between the wave plate and the second polarizer (P2) at θ. In this case, the local field would become right-handed, written as $\mathbf{E}_{\mathrm{R}}=E_{\mathrm{in}}\cos\theta\mathbf{i}+E_{\mathrm{in}}\mathrm{e}^{-\mathrm{i}\frac{\pi}{2}}\sin\theta\mathbf{j}$, where **i** and **j** are the same unit vectors as we defined before. (Note that **i** and **j** do not change when rotating the orientation of the wave plate.) Similar to the previous case, we have

$$E_{\mathrm{o}}^{\mathrm{R}}=\mathrm{e}^{-\mathrm{i}\frac{\pi}{2}}E_{\mathrm{in}}(-\mathrm{e}^{-\mathrm{i}\frac{\pi}{2}}\cos\theta\sin\theta+\sin\theta\cos\theta)=(1-\mathrm{i})E_{\mathrm{in}}\sin\theta\cos\theta \quad (5)$$

$$E_{\mathrm{s}}^{\mathrm{R}}=\beta\tilde{\chi}E_{\mathrm{in}}\left[\mathrm{e}^{-\mathrm{i}\frac{\pi}{2}}\cos\theta\cos\left(\frac{\pi}{4}+\theta\right)\cos\frac{\pi}{4}+\sin\theta\cos\left(\frac{\pi}{4}-\theta\right)\cos\frac{\pi}{4}\right]. \quad (6)$$

Where E_{o}^{R} represents the transmitting light field after P2, $E_{\mathrm{s}}^{\mathrm{R}}$ represents the nanotube scattering field after P2 in this case. Then we could derive

$$\frac{\Delta T_{\mathrm{R}}}{T}=\frac{2\mathrm{Re}\left(E_{\mathrm{o}}^{\mathrm{R}}(E_{\mathrm{s}}^{\mathrm{R}})^{*}\right)}{\left|E_{\mathrm{o}}^{\mathrm{R}}\right|^{2}}=\frac{\beta\chi_{1}}{\sin2\theta}+\frac{\beta\chi_{2}(\cos2\theta-\sin2\theta)}{\sin2\theta}\approx\frac{\beta(\chi_{2}+\chi_{1})}{\sin2\theta}\ . \quad (7)$$

Supplementary Note 2. Determine the efficiency constant β

The supercontinuum with a Gaussian spatial profile can be described as

$$E(x,y) = E_0 e^{-\frac{(x-x_c)^2+(y-y_c)^2}{R^2}}. \tag{8}$$

Where x_c and y_c are the coordinates of the center position of the focus and R is a measure of the beam size. So energy density function of laser can be described as

$$I(x,y) = I_0 e^{-\frac{2(x-x_c)^2+2(y-y_c)^2}{R^2}}. \tag{9}$$

For a 1D nanotube (with a small diameter d) along y direction and positioned at x, the ratio between the total scattering intensity and incident light intensity over the nanotube length is[2-5]

$$\frac{(E_{\mathrm{NT}}^{\mathrm{i}})^2}{(E_{\mathrm{i}})^2} = \frac{\int_{-\infty}^{\infty} \eta_1 \cdot \sigma \cdot e^{-\frac{2(x-x_c)^2+2(y-y_c)^2}{R^2}} dy \cdot \eta_2}{\int_{-\infty}^{\infty}\int_{-\infty}^{\infty} e^{-\frac{2(x-x_c)^2+2(y-y_c)^2}{R^2}} dxdy}$$

$$\approx \frac{e^{-\frac{2(x-x_c)^2}{R^2}} \cdot \sigma}{\frac{\sqrt{2\pi}R}{2}} \cdot \eta_1 \cdot \eta_2 = \eta_1 \cdot \eta_2 \cdot \sqrt{\frac{2}{\pi}} \cdot \frac{\sigma}{R} \cdot e^{-\frac{2(x-x_c)^2}{R^2}}. \tag{10}$$

Where η_1 is the excitation efficiency (about $1/\sqrt{2}$), η_2 is the collection efficiency of objective, σ is the scattering cross-section per unit length[4, 5] and it has the form of

$$\sigma(\omega) = \frac{\pi^2}{64c^3} d^4 \omega^3 |\tilde{\chi}(\omega)|^2. \tag{11}$$

Here ω is the angular frequency of light, c is the speed of light and d is the diameter of nanotube. With above equations, we obtain quantitative value of the β as

$$\beta = \sqrt{\frac{\pi^{3/2} d^4 \omega^3 \ \eta_1 \eta_2}{32\sqrt{2} c^3 R} e^{-\frac{2(x-x_c)^2}{R^2}}}. \tag{12}$$

Supplementary Note 3. Extract real and imaginary part of complex optical susceptibility from the reflection homodyne modulation signal.

The substrate-reflected field ($E_{\mathrm{o}}^{\mathrm{L}}$) and nanotube-scattered field ($E_{\mathrm{s}}^{\mathrm{L}}$) would be

$$E_{\mathrm{o}}^{\mathrm{L}} = rE_{\mathrm{in}}\mathrm{e}^{-\mathrm{i}\frac{\pi}{2}}(\mathrm{e}^{-\mathrm{i}\frac{\pi}{2}}\cos\theta\sin\theta - \sin\theta\cos\theta) = r(\mathrm{i}-1)E_{\mathrm{in}}\sin\theta\cos\theta \tag{13}$$

$$E_{\mathrm{s}}^{\mathrm{L}} = (1+r)^2\beta\tilde{\chi}E_{\mathrm{in}}\left[\mathrm{e}^{-\mathrm{i}\frac{\pi}{2}}\sin\theta\cos\left(\frac{\pi}{4}-\theta\right)\cos\frac{\pi}{4} + \cos\theta\cos\left(\frac{\pi}{4}+\theta\right)\cos\frac{\pi}{4}\right]. \tag{14}$$

Where r represents the reflection coefficient calculated from Fresnel equations and $1+r$ represents the local field experienced by the nanotube.

Then we could derive

$$\frac{\Delta T_{\mathrm{L}}}{T} = \frac{2\mathrm{Re}\left(E_{\mathrm{o}}^{\mathrm{L}}(E_{\mathrm{s}}^{\mathrm{L}})^*\right)}{\left|E_{\mathrm{o}}^{\mathrm{L}}\right|^2} = \frac{(1+r)^2}{r}\left[\frac{\beta\chi_2(\cos2\theta-\sin2\theta)}{\sin2\theta} - \frac{\beta\chi_1}{\sin2\theta}\right]$$

$$\approx \frac{(1+r)^2}{r}\frac{\beta(\chi_2-\chi_1)}{\sin2\theta}. \tag{15}$$

$$\frac{\Delta T_{\mathrm{R}}}{T} = \frac{2\mathrm{Re}\left(E_{\mathrm{o}}^{\mathrm{R}}(E_{\mathrm{s}}^{\mathrm{R}})^*\right)}{\left|E_{\mathrm{o}}^{\mathrm{R}}\right|^2} = \frac{(1+r)^2}{r}\left[\frac{\beta\chi_1}{\sin2\theta} + \frac{\beta\chi_2(\cos2\theta-\sin2\theta)}{\sin2\theta}\right]$$

$$\approx \frac{(1+r)^2}{r}\frac{\beta(\chi_2+\chi_1)}{\sin2\theta}. \tag{16}$$

Thus, we could see that the optical signal will be universally increased by $(1+r)^2/r$ from transmission to reflection configuration.

Supplementary Note 4. Estimate the sensitivity of our technique

This technique is based on the manipulation of interference between incident left- (right-) handed elliptically polarized light and materials' scattering light, physically this technique is not limited to CNTs, but establishes a general analytical means for the entire class of 1D materials. The only limitation should come from the signal-to-noise level of other 1D systems. Here we consider the detection limit of our technique and estimate how far our technique can go for other 1D materials with possible smaller signal. Firstly, according to the performance of our detector (linear CCD, Imaging Solution Group, LW ELIS-1024a-1394, 14 bit), the minimum contrast signal that can be measured is about 10^{-4} (the average of sufficient data to minimize the random noise is needed). Secondly, considering ~20-100 times enhancement of the technique based on polarization manipulation, the detection limit of our technique could be ~10^{-6}.

Supplementary References:

1. Liu, K., Deslippe, J., Xiao, F., Capaz, R.B., Hong, X. et al. An atlas of carbon nanotube optical transitions. *Nat Nanotechnol* **7**, 325-329 (2012).
2. Liu, K.H., Hong, X.P., Choi, S., Jin, C.H., Capaz, R.B. et al. Systematic determination of absolute absorption cross-section of individual carbon nanotubes. *PNAS* **111**, 7564-7569 (2014).
3. Lindfors, K., Kalkbrenner, T., Stoller, P. & Sandoghdar, V. Detection and spectroscopy of gold nanoparticles using supercontinuum white light confocal microscopy. *Phys Rev Lett* **93**, 037401 (2004).
4. Heinz, T.F. Rayleigh scatteing spectroscopy. *Carbon Nanotubes* **111**, 353-369 (Springer, 2007).
5. Malic, E., Hirtschulz, M., Milde, F., Wu, Y., Maultzsch, J. et al. Theory of Rayleigh scattering from metallic carbon nanotubes. *Phys Rev B* **77**, 045432 (2008).
